# Investigation of symptoms in emergency medical calls by patients treated with thrombectomy: a retrospective study in Western Norway

**DOI:** 10.1186/s12245-026-01121-4

**Published:** 2026-01-21

**Authors:** Mikal Olsen Smaadal, Emil Iversen, Guttorm Brattebø

**Affiliations:** 1 The Faculty of Medicine and Dentistry, Department of Clinical Medicine, University of Bergen, Bergen, Norway; 2 Norwegian National Advisory Unit on Emergency Medical Communication (KoKom), Haukeland University Hospital, Bergen, Norway; 3 Department of Anaesthesia and Intensive Care, Haukeland University Hospital, Bergen, Norway

**Keywords:** Stroke, Neurology, Emergency service, Hospital, Emergency medical dispatch

## Abstract

**Background:**

Patients with large vessel occlusion (LVO) may benefit from endovascular thrombectomy (EVT). Early identification and potential transfer to a comprehensive stroke centre can be advantageous, depending on geographical and logistical factors. This study aimed to describe symptoms reported by the caller for patients during emergency medical calls who had acute stroke and later underwent EVT, aiming to guide further research.

**Methods:**

This retrospective descriptive study enrolled patients treated with EVT at Haukeland University Hospital (HUH) who had a primary emergency call to the Bergen Emergency Medical Communication Centre (EMCC) in 2019 and 2021. Patients undergoing EVT were included if they made their first call to the Bergen EMCC. Symptoms presented during the emergency call were quantified and categorised.

**Results:**

Sixty-seven patients were treated with EVT and had their emergency call audio logs recorded at the Bergen EMCC. The most frequently mentioned symptom was speech disturbance (79% of the calls), with 75% of these cases being mentioned spontaneously. Patients were reported conscious in 60% of the calls, 43% had facial palsy, and 36% were reported as having fallen or being found lying on the floor. Emergency medical dispatchers (EMDs) asked about at least one ‘talk, smile, raise arms’ or Face-Arm-Speech-Time (FAST) symptom in 57% of the calls, with questions about speech being the most common (55%).

**Conclusions:**

During emergency medical calls for acute stroke, speech disturbance was the most commonly reported symptom in patients with stroke. A substantial discrepancy was found between the recognition of facial palsy by callers, and that noted in the emergency department (ED), with callers not identifying it as often as the ED. These findings might emphasise the need for enhanced training of emergency medical dispatchers (EMDs) to better recognise and report key stroke symptoms during emergency calls; additionally, they highlight symptoms of particular relevance in emergency calls for patients who are eligible for EVT, an area that should be explored in future research.

## Background

Each year, over 10,000 people suffer from acute stroke in Norway, with approximately 85% experiencing ischemic strokes and 15% haemorrhagic strokes [1]. National guidelines for the treatment of stroke mandate the use of intravenous thrombolysis (IVT) and endovascular thrombectomy (EVT). These treatments are often combined, and in 2023, 65% of all patients undergoing EVT underwent IVT before the procedure [2]. A cost-effectiveness analysis conducted by the Norwegian Department of Public Health reviewed the treatments for large vessel occlusions (LVO) and concluded that EVT in combination with IVT yielded more quality-adjusted life years compared to IVT alone [3].

Originally designed for use in internal carotid artery (ICA) and M1 and M2 occlusions, EVT is primarily used to treat LVOs [4, 5]. A national report published in 2023 documented that 86% of all patients treated with EVT had an occlusion of the ICA, M1, or M2 [2].

Several studies indicate that, in ischaemic stroke, a higher National Institutes of Health Stroke Scale (NIHSS) score is predictive of large vessel occlusions (LVOs). Conversely, differentiating LVOs from more distal occlusions is challenging in patients with medium to lower NIHSS scores [6–8]. Typical LVO symptoms also depend on the proximity of the occlusion. A few studies have examined LVO presentation in the emergency dispatch setting. A Finnish study reported speech disturbance (80%) as the most frequent symptom, and other studies from the ED reported features including head or gaze deviation and cortical signs such as aphasia and neglect, often in combination with motor deficits [9–12]. A French study suggested a new LVO stroke scale for the dispatch setting, but per now, there is no consensus on how detection of LVO in medical emergency calls can be increased [13].

In Norway, eight comprehensive stroke centres perform EVT, and the distances between them are considerable. Hence, patients who could benefit from EVT should be transported directly to the appropriate hospital [14]. Emergency medical calls to 113 in Norway are answered by emergency medical personnel at the Emergency Medical Communication Centre (EMCC), primarily nurses, who decide whether to dispatch an ambulance. Although residents have a primary hospital based on their home address, EMCC or ambulance staff may redirect patients to another hospital if an LVO is suspected and EVT may be needed.

A 2016 meta-analysis found that EVT led to better 90-day disability outcomes, as measured by the modified Rankin Scale (mRS), compared to best medical treatment. The benefit was especially notable in patients over 80 years and those treated more than 300 min after symptom onset [15]. Hence, it is desirable that EMCC recognise and correctly triage patients with stroke symptoms who can be transported directly to a comprehensive stroke centre. This study aimed to examine the symptoms reported during medical emergency calls for patients who later underwent EVT, with the goal of exploring this area further to guide future research endeavours.

## Methods

### Setting

In this retrospective cohort study, we examined data extracted from medical emergency calls made to the Bergen EMCC, which serves the city of Bergen and its surrounding municipalities, with a total population of 460,000 [16]. Bergen EMCC receives approximately 55,000 medical emergency calls annually [17]. The patients included in this study underwent EVT in 2019 and 2021 at Haukeland University Hospital (HUH), which is Bergen Health Trust’s only comprehensive stroke centre. The patients were either transported directly to HUH or transferred from other primary stroke centres within its catchment area.

In Norway, local out-of-hours emergency clinics are staffed by general practitioners who treat patients with acute illnesses. A local medical communication centre (LMCC) connected to these clinics handles medical calls from patients with less urgent symptoms than those managed by the EMCC. There are 169 local emergency clinics and 94 LMCCs across Norway [18]. The LMCCs are responsible for selecting patients who can visit the local emergency clinic, whereas some patients receive only medical advice on the phone. In cases where the LMCC operator determines that a patient requires urgent care, the call may be redirected to the EMCC for ambulance dispatch.

All 16 EMCCs in Norway use the Norwegian Index for Emergency Medical Assistance (Index), a decision-support tool originally adapted from the Seattle guidelines of 1994 [19]. Its primary purpose is to support the EMDs in uncovering symptoms and thus decide the urgency and the need for ambulance dispatch. For acute stroke, the Index includes a dedicated section on ‘stroke symptoms’, where each stroke symptom has a given criteria number. The section incorporates questions whether the patient can talk, smile or raise their arms (TSR), derived from the FAST examination and also promoted through public awareness campaigns [20, 21]. It also questions more subtle symptoms of stroke, such as dizziness and confusion. Aphasia and dysarthria are mentioned but not clearly defined, and guidance on speech difficulties relies on non-medical terms such as ‘slurred speech’, ‘mumbling’, or ‘difficulty finding words’. Similarly, facial palsy is addressed by asking whether the patient ‘can smile’ or appears to have a ‘crooked face’ [19]. However, the Index does not provide support in identifying whether the stroke may represent a LVO suitable for EVT.

## Data extraction

We identified patients who underwent EVT at HUH in 2019 and 2021 from a dataset provided by the Norwegian Stroke Registry (NSR). The NSR is an obligatory national quality registry that covers 86% of patients with stroke in Norway [1]. It includes demographics and symptoms described upon admission by the doctor treating the patient in the ED. Subsequently, one of the researchers (MS) extracted audio files from the medical emergency calls using the audio-logging program in Bergen EMCC (NICE Inform, [NICE], Israel). The audio files were transcribed and extracted between March 2022 and November 2022. One researcher (MS) reviewed all audio files from the emergency calls and determined which patients to include. Patients who underwent EVT were included at the start of the study. Only patients with their first call to the Bergen EMCC were included (i.e., those with the first call to the LMCC were excluded). Transfers, whether inter- or intra-hospital, were excluded if the onset of symptoms occurred in the hospital, as data on primary medical emergency calls were not available. As part of a subproject, we already had access to the 2019 patient cohort. We included patients from 2021, but not from 2020, as data older than three years at the time of extraction were automatically deleted due to local privacy regulations, thereby eliminating the risk of data loss during the extraction-phase.

## Symptom categorisation and analysis

The transcripts of the audio logs were read, and the calls were reviewed multiple times. The symptoms were categorised and quantified. To establish a baseline for categorisation, five audio logs were initially reviewed, supported by symptoms listed in both the NIHSS and Index. This was carried out as objectively as possible, and symptoms were noted only if the caller mentioned them spontaneously or in response to a question asked by the EMD. Similar to other studies, terms such as ‘lying on the floor’ were categorised as having sustained a ‘fall’, while those having trouble speaking (aphasia or dysarthria) were categorised as having a ‘speech disturbance’. Issues with walking or standing were included in the ‘motoric leg’ category [22]. The frequencies of the symptoms were then analysed and compared using descriptive statistics. All symptoms were noted and placed under the most appropriate category. Since callers to the EMCC do not use medical abbreviations or medical terms, symptoms (e.g. confused, sweating, stiffness, altered behaviour pattern, breathing difficulties) that did not match NIHSS and Index symptoms were placed under the ‘other symptoms’ category. Visual disturbances were stratified after data collection when it became evident that staring was a recurrent visual symptom.

## Patient and public involvement

None.

## Ethics

This study was approved by the Regional Committee for Medical and Health Research Ethics (REK Vest, No. 2020-108573). All patients included in the study received a letter, granting them the right to withdraw from the study at any time.

## Results

We identified 115 patients from the NSR who underwent EVT at HUH during two years. After reviewing their calls, 69 patients were included in the study. Two additional patients were excluded because the caller was not at the same location as the patient and thus, no symptoms were described. Of the 1231 patients with stroke in 2019 and 2021, 9.3% (*n* = 115) underwent EVT, and 5.4% (*n* = 67) were included in this study.

**Fig. 1:**
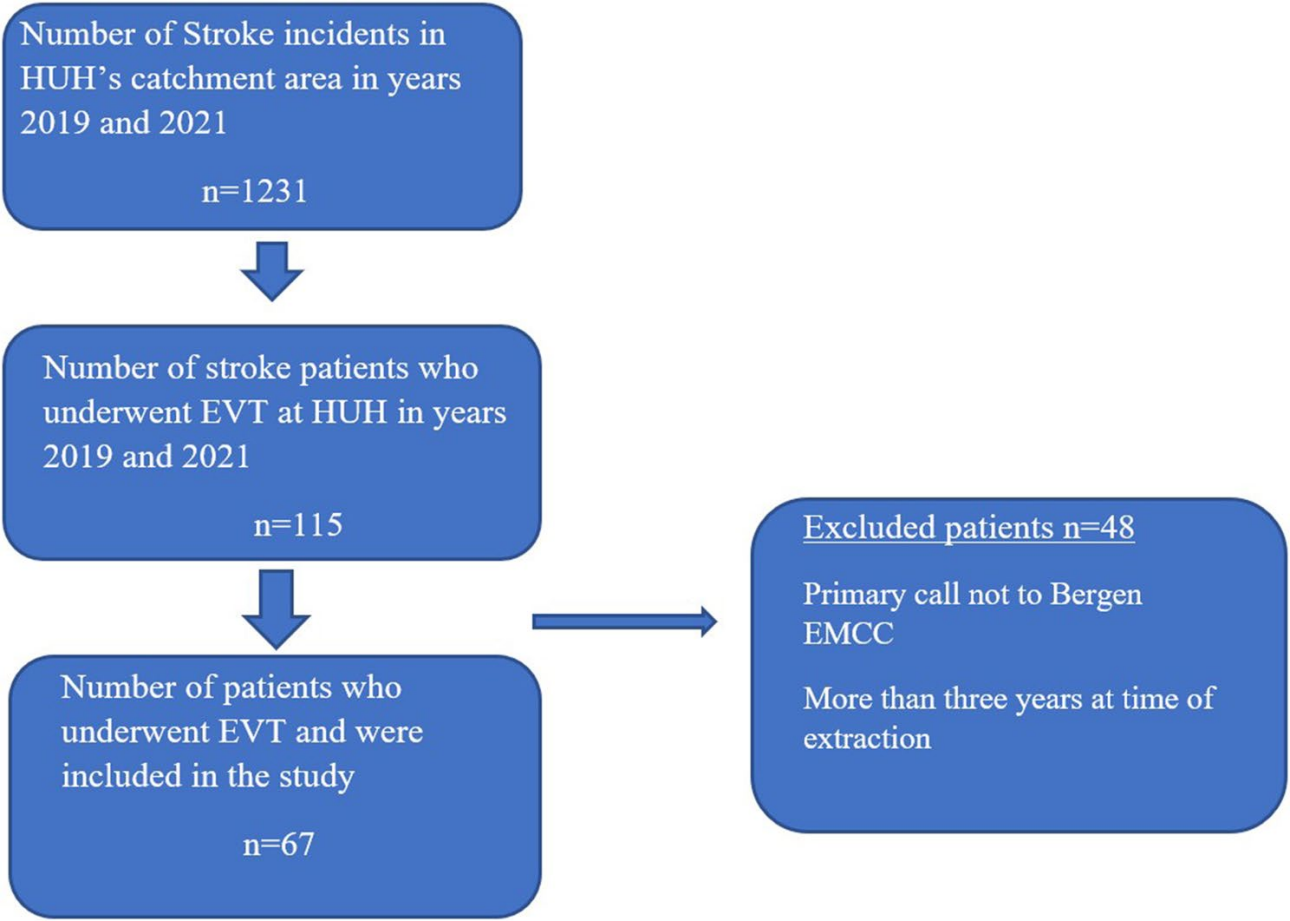
Patient flowchart

In total, 67 patients were included in this study (Fig. 1). The median age was 77 years (range 32–94 years). Stratified by year, the median ages in 2019 and 2021 were 81 and 72 years, respectively. The median ages for women and men were 88 and 79.5 years in 2019, and 81 and 69 years in 2021, respectively. A total of 19% (*n* = 13) of the patients experienced a ‘wake-up-stroke’ according to the NSR. Of the included patients, 57% were male, and 69% (*n* = 46) were treated with IVT before EVT.

## Symptoms

Among the symptoms reported, speech disturbance was the most frequent, noted in 79% (*n* = 53) of the calls. This was followed by mentions of awake status in 60% (*n* = 40) of the calls and facial palsy in 43% (*n* = 29). In 36% (*n* = 24) of the calls, the patient was reported as having fallen or being found lying on the floor (Table 1). The combination of speech disturbance and facial palsy was the most recurrent, occurring in 79% of the calls. According to NSR data, 93% (*n* = 62) of patients had dysarthria or aphasia.

After stratifying speech disturbance into the most common description from callers, “no speech/can’t talk/no verbal contact” was the most prevalent, occurring in 40% (*n* = 21/53) of the cases. “Slurred speech” was mentioned in 17% (*n* = 9/53), “talks sentences that don’t make sense” in 13% (*n* = 7/53) of the cases. “Mumbling” was mentioned in 11% (*n* = 6/53). Other less prevalent descriptions were “incoherent speech” or “can’t form words”. In some of the cases the caller mentioned “speech difficulties” not described further.

**Table 1:**
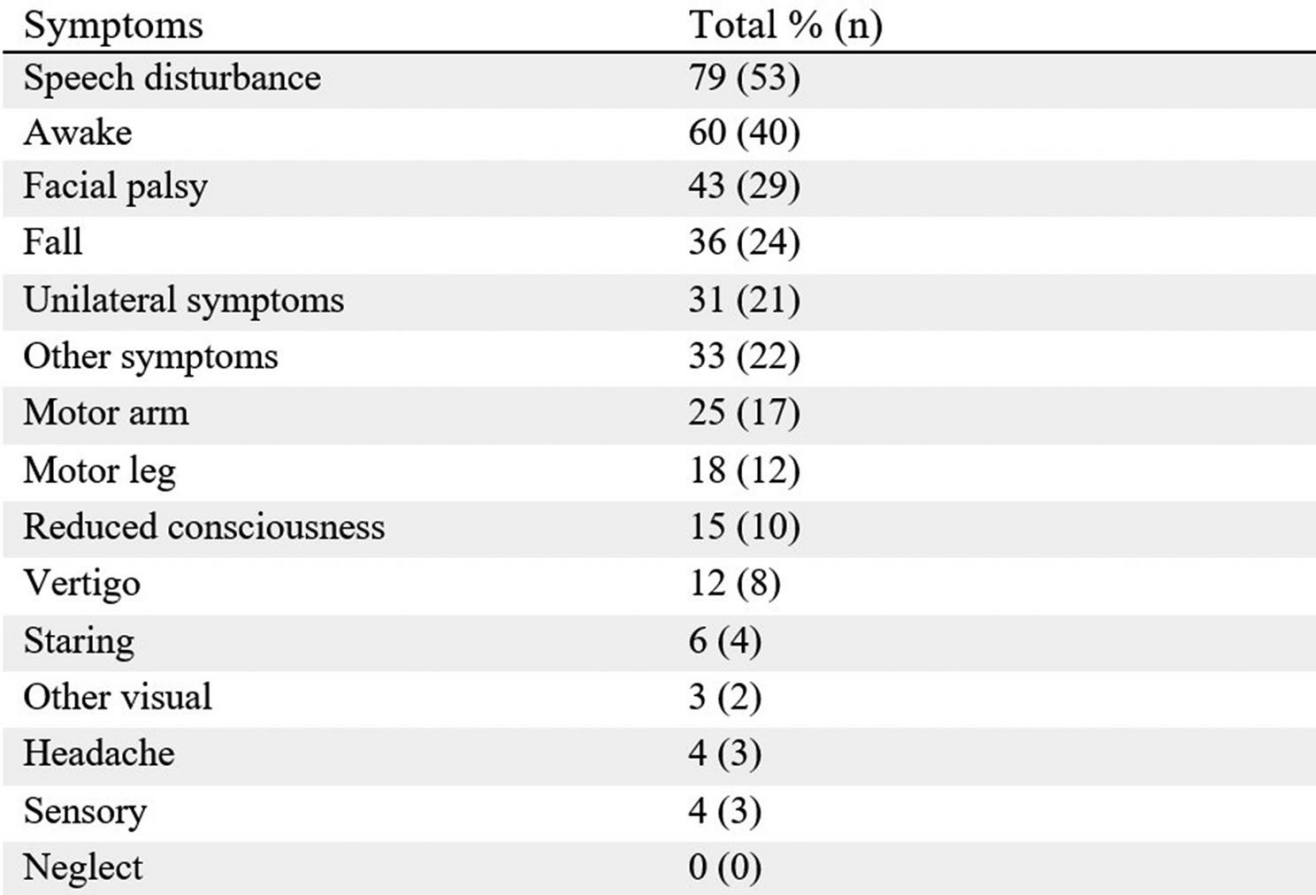
List of symptoms presented in the emergency medical calls

## Spontaneous reporting of symptoms versus reporting upon questioning

During the calls, some symptoms were reported spontaneously by the caller, while others were elicited through direct questioning by the EMCC. Of the TSR or FAST symptoms used in the Index, speech disturbance was mentioned spontaneously by the caller in 75% (*n* = 40/53) of the calls. In addition, 96% (*n* = 64/67) of the patients in the ED had facial palsy, while only 43% (*n* = 29/67) of the callers reported facial palsy (76% of these mentioned it spontaneously). In only 25% (*n* = 17/67) of the calls, problems lifting the arms and/or loss of power in the arms were mentioned (35% of these mentioned it spontaneously). The EMCC asked about at least one TSR/FAST symptom in 57% (*n* = 38/67) of the calls. In this group, the most frequently asked question by the EMCC concerned speech (55%; *n* = 21/38), followed by facial asymmetry (34%; *n* = 13/38), difficulty lifting arms (32%; *n* = 12/38), and lack of pinching-power in hands (24%; *n* = 9/38). Questions about the ability to smile were asked in 13% (*n* = 5/38) of the calls in this group. In only 3% (*n* = 2/67) of the calls, the dispatcher asked about all the TSR/FAST symptoms. In 52% of the calls (*n* = 35/67), the term ‘stroke’ was introduced spontaneously by the caller, whereas in 16% (*n* = 11/67), it was introduced by the EMCC either through direct mention or inquiry. For each call, only the first mention of ‘stroke’ was registered. In the remaining calls, stroke was never mentioned.

### NSR data

In summarising the NSR data from 2019 to 2021, 78% (*n* = 52) of patients were found to have dysarthria when examined by a medical doctor in the ED, and aphasia was observed in 66% (*n* = 44) of ED patients. Neglect was present in 45% (*n* = 30), while 34% (*n* = 23) had a restricted visual field, and 13% (*n* = 9) had double vision.

### Transport methods

Of the 67 patients included, transport methods were recorded for 65 in the NSR. Five patients (8%) were transported to the hospital by air ambulance, while the rest were transported by car ambulance. Furthermore, 81% were transported directly to a comprehensive stroke centre, while 19% were initially taken to a primary stroke centre and later transferred to a comprehensive stroke centre.

## Discussion

In this retrospective descriptive study of 67 emergency medical calls from stroke patients who were treated with EVT, we found that speech disturbance was the most frequently mentioned symptom, reported in 79% of the calls. A Finnish study reported similar findings in patients with LVO [12]. Other studies investigating strokes treated with both IVT and EVT reported speech disturbance as a common symptom, but with a lower prevalence (2–54%), depending on the country and year of study [22–24]. It is possible that patients with LVOs experience more severe speech disturbances than those with smaller occlusions, which the caller may easily notice during emergency calls.

Interestingly, when comparing NSR data with our audio log material, it became evident that speech disturbances such as dysarthria or aphasia were mentioned in the majority of emergency medical calls (*n* = 53/67) but observed in slightly more cases upon arrival in the ED (*n* = 62/67). There might be several reasons for why more patients had speech disturbances in the ED. One explanation might be that the examining doctor uses a more structural examination and can physically see the patient. Other reasons might be that the dispatchers main focus is getting the ambulance to the patients as fast as possible, or because the patient’s symptoms are evolving.

Although multiple studies have demonstrated a high prevalence of cortical symptoms like aphasia in patients with LVOs, establishing this diagnosis at the level of emergency dispatch remains challenging [10, 11]. Callers, who are often not healthcare professionals, frequently describe symptoms in non-medical terms. For example, a report of the patient ‘not being able to talk’ may refer either to complete loss of speech or to impaired articulation. Similarly, descriptions such as ‘slurred speech’ may suggest dysarthria, whereas statements like ‘just talking nonsense’ may, upon further clarification, indicate the presence of aphasia, as the patient produces sentences that lack meaningful content. Our study found that callers most often mentioned that the patient ‘could not talk’, which is supported by the Finnish study [12].

In only 60% of the calls, it was clarified whether the patient was awake. In the remaining calls this was addressed indirectly, for instance if the caller mentioned a ‘talking patient’ or ‘reduced function of left foot’.

Although facial palsy and speech disturbances often co-occur, their symptoms may overlap. Symptoms during emergency medical calls and their definitions are subjective to the callers. Therefore, if a patient’s facial palsy impairs speech, it is challenging to distinguish between facial palsy and speech disturbances (related to cortical symptoms involved in aphasia) from audio logs alone, unless each symptom is asked about separately, and both symptoms are noted. The dispatchers asked about facial palsy in 13 of the calls, often using the wording ‘crooked face’ or ‘can the patient smile?’, this is also subjective to the caller who may never have seen facial palsy before. The use of video during emergency medical calls might be beneficial for this assessment [25]. Stroke is a dynamic condition that worsens without treatment, which could explain the substantial discrepancy between the recognition of facial palsy by callers compared to its recognition in the ED (43% vs. 96%) [26, 27].

Callers were asked about at least one TSR/FAST symptom in 57% of the calls, with questions about speech being the most frequently asked. However, in the majority of calls (75%), issues with talking or speech disturbances were mentioned spontaneously by the caller. Therefore, the EMDs did not need to ask specifically about them. This suggests that mass media interventions focusing on TSR symptoms may have increased public awareness of stroke symptoms. This point is partially supported by an article published in 2016 [20]. Moreover, patients with LVOs often present with severe symptoms, including speech disturbances (aphasia or dysarthria) [10].

### Strengths and limitations

There are several strengths to this study. The audio logs were both listen to and transcribed by someone else than the examiner (MS), reducing the confirmation bias. Only calls made directly to the EMCC were included, while calls routed through the LMCC were excluded. This ensured that the callers’ descriptions reflected their initial, unprompted observations, without being influenced by prior interaction with an LMCC dispatcher. Initially, only one examiner (MS) analysed the transcribed audio files. However, several audio logs were also reviewed by EI, and all authors discussed the data analysis afterwards. There are also several limitations to this study. Although all patients treated with EVT in 2019 and 2021 with an audio record from the Bergen EMCC were included, the sample size was relatively small. The study lacked control data beyond the data from the Norwegian Stroke Registry. This also resulted in the use of simple descriptive statistics.

## Conclusion

We found that speech disturbance, was the most frequently mentioned symptom in emergency medical calls involving patients with stroke later treated with EVT. Facial palsy was mentioned spontaneously more often than other symptoms. We also found the discrepancy between the recognition of facial palsy during emergency medical calls and its observation in the ED notable. Balancing the assessment of ambulance priority with the collection of an adequate medical history poses a significant challenge for the dispatcher. This underscores the need for further research to improve the identification of LVO during emergency medical calls.

## Data Availability

Additional data may be available upon reasonable request to the corresponding author.

## Abbreviations

EVT: Endovascular thrombectomy
HUH: Haukeland University Hospital
EMCC: Emergency Medical Communication Centre
EMDs: Emergency medical dispatchers
FAST: Face-Arm-Speech-Time
ED: Emergency department
IVT: Intravenous thrombolysis
LVO: Large vessel occlusions
ICA: Internal carotid artery
mRS: Modified Rankin Scale
LMCC: Local medical communication centre
TSR: Talk, smile, or raise their arms
NSR: Norwegian Stroke Registry
NIHSS: National Institutes of Health Stroke Scale
